# Performance of a New HPV Cervi-Collect Collection and Transportation Kit

**DOI:** 10.1155/2012/503432

**Published:** 2011-11-14

**Authors:** M. Chernesky, S. Huang, D. Jang, B. Erickson, J. Salituro, H. Engel, J. Gilchrist, P. Neuscheler, W. B. Mak, K. Abravaya

**Affiliations:** ^1^St. Joseph's Healthcare Hamilton, McMaster University, Hamilton, ON, Canada L8N 4A6; ^2^Abbott Molecular Inc., Des Plaines, IL 60018-3315, USA; ^3^Abbott Diagnostics, 65205 Wiesbaden, Germany

## Abstract

*Background*. Liquid-based Pap (L-Pap) media are used for Pap and human papillomavirus (HPV) testing. *Objectives*. To compare RealTi*m*e High Risk (HR) HPV testing of a new collection kit (Cervi-Collect) and PreservCyt L-Pap specimens. To determine ease of use and safety of Cervi-Collect. *Methods*. L-Pap samples (*n* = 203) were tested with HC2 and RealTi*m*e HR HPV and Cervi-Collect with RealTi*m*e HR HPV. Discordant samples were genotyped. *Results*. L-Pap and Cervi-Collect specimens tested by RealTi*m*e HR HPV showed 93.1% agreement (Kappa 0.86). RealTi*m*e HR HPV and HC2 on L-Pap had 90.3% agreement (Kappa 0.80). RealTi*m*e HR HPV on Cervi-Collect and HC2 on L-Pap showed 88.2% agreement (Kappa 0.76). Sixteen of 21 samples which were HC2 negative and RealTi*m*e HR HPV positive on L-Pap or Cervi-Collect contained HR HPV genotypes. Eleven healthcare collectors were in strong agreement on a usability and safety questionnaire. *Conclusion*. Cervi-Collect samples were easy to collect and showed strong agreement with L-Pap samples tested with RealTi*m*e HR HPV or HC2.

## 1. Introduction

High-risk human papillomaviruses (HR HPV) are a major cause of cervical cancer [1]. HR HPV testing either adjunctively with cytology or as the primary screening test has shown increased sensitivity for detecting CIN2+ precancerous lesions when compared with Pap testing alone [2]. ThinPrep PreservCyt Solution and SurePath Preservative Fluid are transportation and storage media enabling Pap and HPV testing. PreservCyt liquid-based (L-Pap) medium has been validated with the Abbott RealTi*m*e HR HPV assay. In cases where Pap testing is performed using non-L-Pap samples or HPV testing is performed as the primary screening method, a cervical specimen is collected for HPV testing. A collection brush and transportation medium kit (Cervi-Collect) was designed by Abbott Molecular for testing with the Abbott RealTi*m*e HR HPV assay. The principles and analytical performance of this assay have been described [3], and there are several reports comparing it to HC2 in archived samples [4–6] and to various DNA and RNA detection methods in L-Pap samples [7–9]. 

The aims were as follows: (a) to compare the performance of the RealTi*m*e HR HPV assay by testing Cervi-Collect and PreservCyt L-Pap specimens, (b) to compare the RealTi*m*e HR HPV and HC2 assays on L-Pap specimens, (c) to test discordant samples in a linear array (LA) assay, and (d) to analyze the strength of agreement of healthcare workers on ease of use and safety of the collection device and its package insert using a questionnaire.

## 2. Material and Methods 

A total of 203 women attending a women's health clinic undergoing a routine gynecological exam or a follow-up exam due to an abnormal Pap or positive HR HPV test signed consent to have 2 cervical specimens collected: the first was collected with a Cervex-Brush (Rovers Medical devices, Oss, The Netherlands) and placed into an L-Pap PreservCyt collection medium tube (Hologic Inc, Marlborough, Mass, USA) and the second was collected with the Cervi-Collect brush and placed into a Cervi-Collect transportation tube. Specimen collection was performed according to the respective manufacturers' instructions. The PreservCyt sample was processed for cytology in the Pathology Laboratory at the Juravinski Hospital, Hamilton, ON, Canada, and the remainder of the sample was sent to the Infection Research Laboratory (IRL) at the St. Joseph's Healthcare Hamilton, Hamilton, ON, Canada. Both samples were received within 24 hours in the IRL. 

### 2.1. HC2 Testing

The L-Pap sample was tested for HR HPV with the HC2 test (Qiagen, Gaithersburg, Md, USA) at the IRL according to the package insert. Previous positive and negative clinical samples were included with each run as controls. Samples were scored negative if relative light units/cutoff (RLU/CO) ratios were <1.0, indeterminate when ≥1.0 and <2.5, and positive when ≥2.5. Indeterminate samples were repeated in duplicates: a sample with an RLU/CO ratio ≥1.0 in either replicate was considered positive.

### 2.2. RealTi*m*e HR HPV Testing

The Cervi-Collect sample and one milliliter of the L-Pap sample were packaged and shipped to Abbott Diagnostics in Wiesbaden, Germany, where they were tested in a blinded fashion with the RealTi*m*e HR HPV assay on the Abbott *m*2000 instrument. The automated test procedure consisted of sample preparation, reaction assembly, real-time PCR, and result reporting [3]. During sample preparation using the Abbott *m*2000*sp*, 0.4 mL of sample was processed using the Abbott *m*Sample Preparation System_DNA_ where it was lysed with chaotropic reagents, allowing the DNA to be captured on magnetic microparticles. The bound purified DNA was washed and then eluted. An amplification master mix was created with AmpliTaq Gold enzyme (Roche Molecular Systems Inc., Branchburg, NJ, USA), magnesium chloride, and an oligonucleotide reagent containing primers, probes, and dNTPs. The PCR reaction was then assembled in a 96-well optical reaction plate by combining aliquots of the master mix and the extracted DNA eluate. Thermocycling and fluorescence detection of the amplified products were carried out in the Abbott *m*2000 real-time PCR instrument, and results were automatically reported. The assay detects 14 HR HPV genotypes (16, 18, 31, 33, 35, 39, 45, 51, 52, 56, 58, 59, 66, and 68) with type specific detection for types 16 and 18 and detection of the other 12 non-HPV 16/18 types as a group. A separate detection category of *β*-globin is included as an internal control to validate sample adequacy, DNA recovery, and PCR efficiency. Results for each sample were reported based on all three HPV signals, corresponding to HPV16, HPV 18, and non-HPV 16/18 HR types, as well as the internal control signal. 

### 2.3. LA Testing

Samples which showed discordant results after testing by HC2 and RealTi*m*e HR HPV assays were tested using the LA HPV Genotyping Test (Roche Molecular Systems Inc., Branchburg, NJ, USA) following the manufacturer's protocol. PCR was performed in a final reaction volume of 100 *μ*L containing 50 *μ*L of kit master mix. The genotyping strips were visually interpreted using the HPV reference guide provided in the kit package insert. The same high-risk genotypes represented in the Abbott assay were considered high risk.

### 2.4. Questionnaires

Sample collectors (physicians and nurses) were asked to complete a questionnaire rating whether the product labeling information was adequate and easy to understand in the following areas: the intended use statement, the instructions for safe use, collection, storage, and transport, and limitation of use statement in the package insert. They also evaluated the usability aspects (such as whether the kit package was easy to open, whether the tube cap was easy to take off and replace, and whether any leakage was present) as well as the safety aspects for the collection kit and instructions. In total, eleven questions were answered by each of the eleven collectors. Each question was answered on a scale of 1 to 5, with 5 indicating strong agreement with a statement and 1 if there was strong disagreement. The overall rating across all collectors for each question was calculated as the combined score as a percentage of a maximal score of 55 (i.e., 11 times 5).

### 2.5. Statistical Analysis

Agreement between tests was assessed by kappa statistic (*κ*).

## 3. Results

There was strong agreement between the L-Pap and Cervi-Collect specimens tested by RealTi*m*e HR HPV (Table 1). The positive agreement was 85.7% (84/98), negative agreement was 88.2% (105/119), and overall agreement was 93.1% (189/203) (Kappa 0.86). There were 8 L-Pap samples with insufficient volume for HC2 testing (4 were from patients who were negative in Cervi-Collect and L-Pap samples and 4 were positive in both by RealTi*m*e HR HPV).  Table 2 shows agreement between RealTi*m*e HR HPV and HC2 performed on 195 L-Pap specimens. The assays agreed on 73 positives and 103 negatives. There were 15 samples which were positive by the RealTi*m*e HR HPV test and negative by HC2, and 4 other samples which were positive by HC2 but negative by the RealTi*m*e HR HPV test. The positive agreement was 79.3% (73/92), negative agreement was 84.4% (103/122), and the overall agreement was 90.3% (176/195) (Kappa 0.80). When the RealTi*m*e HR HPV test was performed on Cervi-Collect specimens and HC2 was performed on L-Pap (Table 3), positive agreement was 75.3% (70/93), negative agreement was 81.6% (102/125), and overall agreement was 88.2% (172/195) (Kappa 0.76).

Table 4 summarizes the results of LA testing of 28 discordant samples from the 3 testing strategies (HC2 on L-Pap, RealTi*m*e HR HPV on L-Pap, and RealTi*m*e HR HPV on Cervi-Collect). Samples from 16 of 21 patients with a negative HC2 result and a positive RealTi*m*e HR PCR result obtained either from L-Pap or Cervi-Collect samples contained HR HPV genotypes. Samples from 4 patients (026, 040, C121, and 190) which were positive by HC2 and negative by the RealTi*m*e HR HPV assay in the L-Pap and Cervi-Collect samples contained low-risk HPV genotypes. Three patients (099, C169, and C193), which were HC2 and RealTi*m*e positive in L-Pap but were negative in the Cervi-Collect sample, contained HR genotypes. 

**Figure 1:**
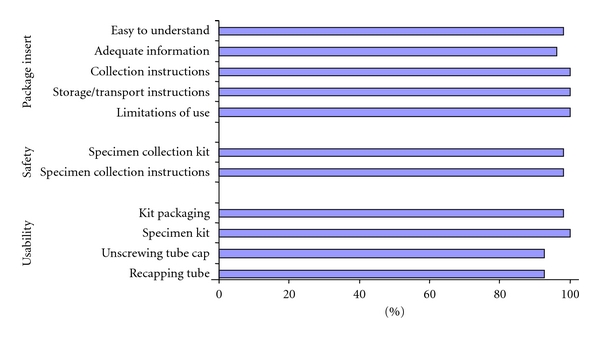
Evaluation of the Cervi-Collect package insert, safety, and usability rated by 11 healthcare collectors. (Each of the eleven questionnaire categories were graded by the collectors, with 5 being the most favorable because of total agreement with the statement. The final rating was based on the combined score from all the collectors as a percentage of the maximal score of 55. E.g., 100% is representative of 11 healthcare collectors giving a combined score of 55).

Figure 1 summarizes the outcomes from the questionnaires. Four of 11 categories received a full score (100%) out of a maximal score of 55 (5 from all 11 collectors), and the other 7 categories were graded at the maximum by most collectors (8 or greater) with an overall rating between 93% and 98%. The lower scores (93%) were recorded in categories for unscrewing and recapping the tube.

## 4. Discussion

The new Cervi-Collect kit compared well to PreservCyt when tested by the RealTi*m*e HR HPV assay (Table 1), showing strong agreement of 93.1% (Kappa = 0.86). Analysis of the 98 samples which were positive in either sample type from Table 1 showed that 27 were positive in the type 16 signal (with or without the non-HPV 16/18 HR HPV signal), 12 were positive in the type 18 signal, 3 were positive in both the HPV 16 and 18 signals, and the rest were positive only in the non-HPV 16/18 HR HPV signal. The higher agreement between the two RealTi*m*e HR HPV results for different transport media compared to that between RealTi*m*e HR HPV and HC2 was mainly due to more positives in agreement (*n* = 84 in Table 1 versus 70 or 73 in Tables 2 and 3, resp.). 

Comparing assays in Tables 2 and 3 showed more cases of HC2 negative/RealTi*m*e HR HPV positive than HC2 positive/RealTi*m*e HR HPV negative samples. These differences are consistent with findings in other studies [7–9] which showed that the RealTi*m*e HR HPV assay detected the same number or more cases of HPV infection than the HC2 test. The HR HPV positive samples that were not detected by the HC2 test contained HR genotypes by the LA test (Table 4). There were 16 patients positive by RealTi*m*e HR HPV in the Cervi-Collect sample and negative by HC2 in the L-Pap sample, 13 of which contained HR HPV by LA testing. Ten of the 13 were also positive by RealTi*m*e HR HPV in the L-Pap samples. Of the total 28 discordant samples, 24 were positive for HPV and 19 showed the presence of HR HPV genotypes by LA testing. Of these 19 samples, 13 Cervi-Collect samples were identified as HR HPV positive by the RealTi*m*e HR HPV assay, 16 L-Pap samples positive by RealTi*m*e HR HPV, and 3 L-Pap samples positive by HC2 (Table 4). The study was not designed to follow patients to colposcopy and biopsy, so one can only speculate what the significance of these additional positive infections would be in predicting precancerous lesions. Examination of the 7 samples positive by HC2 and negative by RealTi*m*e HR HPV on Cervi-Collect revealed 3 samples that were confirmed positive by LA and RealTi*m*e HR HPV on the L-Pap sample. All 3 samples contained a low level of HPV targets as indicated by results from both assays. Because the new collection device was experimental, the L-Pap sample was required to be collected first and the Cervi-Collect brush was used to collect the second sample. Low levels of target, collection order, and analytical sensitivity differences for HC2, RealTi*m*e HR HPV, and LA may contribute to variability of assay comparison. The other 4 samples, only positive by HC2 testing, were shown to contain no HR HPV but a variety of low-risk (LR) genotypes by LA (Table 4, patients 26, 40, C121, and 190). Cross-reactivity of the HC2 test with low-risk HPV genotypes has been reported previously. Sandri et al. [10] showed that low risk genotypes such as HPV types 6, 42, 62, 71, 73, and 81 were found to be reactive in the HR HC2 test. Castle et al. [11] showed that genotypes not targeted in the HR HC2 panel most often testing positive were HPV 82 (80%), HPV 70 (59.1%), and HPV 67 (56.3%).

Analysis of the questionnaire scores (Figure 1) showed that 4 of the eleven categories received the maximum score of 5 by all respondents (55 = 100%). Unscrewing and recapping the tube received scores of 3 or 4 by 3 of the eleven collectors suggesting that these maneuvers may be difficult for some collectors due to a certain degree of dexterity required.

## 5. Conclusion

Because cervical samples may be collected specifically for HPV testing, a system suitable for the collection, transportation, and storage of specimens for the detection of HR HPV DNA by the Abbott RealTi*m*e HR HPV was developed and evaluated. Cervi-Collect was designed to achieve efficient cervical collection, optimal sample stability, and compatibility with the automated sample preparation instrument (Abbott *m*2000*sp*) as the primary input tube. This study demonstrated excellent performance of the Cervi-Collect samples for the detection of HR HPV when tested with RealTi*m*e HR HPV compared with the PreservCyt L-Pap samples tested with RealTi*m*e HR HPV or HC2. The Cervi-Collect samples were not evaluated as a source for cytological examination. Healthcare collectors showed strong agreement with the usability and safety design features of Cervi-Collect and its package insert. Further studies need to be conducted to determine the versatility of this new collection kit for other anatomical sites such as the vagina [12], anus [13], and oropharynx [14] as well as other sexually transmitted infections [15, 16].

## Figures and Tables

**Table 1:** Agreement between Cervi-Collect and PreservCyt L-Pap specimens tested by the Abbott RealTi*m*e HR HPV assay.

		Abbott RealTi*m*e HR HPV with Cervi-Collect	
		+	−
Abbott RealTi*m*e HR HPV with PreservCyt L-Pap	+	84	8
	−	6	105

Positive agreement—85.7% (84/98); Negative agreement—88.2% (105/119); Overall agreement—93.1% (189/203) (kappa = 0.86).

**Table 2:** Correlations between Abbott RealTi*m*e HR HPV and Hybrid Capture 2 with PreservCyt L-Pap Samples.

		Abbott RealTi*m*e HR HPV with PreservCyt L-Pap	
		+	−

Hybrid Capture 2 with PreservCyt L-Pap	+	73	4
	−	15	103

Positive agreement—79.3% (73/92); Negative agreement—84.4% (103/122); Overall agreement—90.3% (176/195) (kappa = 0.80).

**Table 3:** Correlation between Abbott RealTi*m*e HR HPV with Cervi-Collect and Hybrid Capture 2 with PreservCyt L-Pap Samples.

		Abbott RealTi*m*e HR HPV with Cervi-Collect	
		+	−

Hybrid Capture 2 with PreservCyt L-Pap	+	70	7
	−	16	102

Positive Agreement—75.3% (70/93); Negative agreement—81.6% (102/125); Overall agreement—88.2% (172/195) (kappa = 0.76).

**Table 4:** Comparison of discordant samples tested by linear array (LA).

Patient number	RealTi*m*e HR HPV on Cervi-Collect	RealTi*m*e HR HPV on L-Pap	HC2 on L-Pap	HPV genotypes^1^
025	HR HPV	HR HPV	NEG	**59**, **66**, **68**, 81
029	HR HPV	HR HPV	NEG	**45**
058	HR HPV	HR HPV	NEG	**51**
084	HR HPV	HR HPV	NEG	**16**, **18**, **39**, **51**, 54, **66**, CP6108
085	HR HPV	HR HPV	NEG	**51**, **66**
C104	HR HPV	HR HPV	NEG	**31**, 62
C129	HR HPV	HR HPV	NEG	**39**, **66**
C158	HR HPV	HR HPV	NEG	**52**
C156	HPV 18	HPV 18	NEG	**18**, 84
177	HPV 16	HPV 16	NEG	**16**
C112	HR HPV	Not Detected	NEG	NEG
C131	HR HPV	Not Detected	NEG	NEG
C173	HR HPV	Not Detected	NEG	**16**, **59**, 62, 70
C182	HPV 16	Not Detected	NEG	**16**, 40, 53, 55
186	HPV 16	Not Detected	NEG	81, CP6108
C167	HPV 18	Not Detected	NEG	**18**, 42, **73**
081	Not Detected	HR HPV	NEG	**35**, **52**, **59**
C128	Not Detected	HR HPV	NEG	**18**
099	Not Detected	HR HPV	POS	**51**, 54, **56**, 62
C169	Not Detected	HR HPV	POS	**56**, 84
060	Not Detected	HR HPV	NEG	NEG
070	Not Detected	HPV 16	NEG	**16**
095	Not Detected	HPV 16	NEG	NEG
C193	Not Detected	HPV 16	POS	**16**
026	Not Detected	Not Detected	POS	IS39
040	Not Detected	Not Detected	POS	40, 53, CP6108
C121	Not Detected	Not Detected	POS	53
190	Not Detected	Not Detected	POS	81, 84

^1^High-risk HPV genotypes are bolded.
